# Is ischemic stroke more severe in patients with migraine vs. other headache types?

**DOI:** 10.1186/1129-2377-14-S1-P144

**Published:** 2013-02-21

**Authors:** V Maticiuc, I Moldovanu, S Odobescu, O Grosu

**Affiliations:** 1 National Institute of Neurology and Neurosurgery, Moldova, Republic of

## Background

Migraine (Mg) is recognized as a cardiovascular risk factor and connection with stroke (S) is well known, especially for Mg with aura.

## Objectives

To evaluate the influence of Mg on the severity of S compared to other types of headache and the relationship between Mg and S (according to Welsh criteria).

## Material and methods

The study included 134 pts with S and headache. After statistical adjustment for age, gender and affected vascular brain system, 2 groups were formed: I - 15 pts with S and Mg, II- 16 pts with S and other types of headache. All patients had confirmation of S on the cerebral CT or IRM 1,5T. Headache was assessed according to ICHD-II (2004), Welch criteria were applied for the relationship between Mg and S. The Rankin score for S disability was preformed.

## Results

The I group (8 women and 7 men) mean age 49, 53 ± 11.59 years: 9 pts had Mg with aura (MA) and 6 pts - without aura (MO). The second group (8 women and 8 men) mean age 53.87± 6.8 years, 12 pts- tension type headache, 3 pts - headache attributed to HTA and 1 pts - unclassified. There were no differences between groups on main vascular risk factors (blood pressure, cholesterol level and ischemic cardiopathy). According to Welch criteria 2 pts ( 13.3%) had class I category of relationship between Mg and S – “coexisting migraine and stroke”- S occurs in a patient with Mg, but not during an attack, 9 pts (60%) had class IIIB ” migraine-induced stroke; with risk factors” - MA produces S in the presence of another risk factor and 4 pts ( 26.6%) had the class IV - “uncertain” history of MO and S during a Mg attack. The Rankin disability scale was significantly worse in S and Mg group (3.0 ± 1.0 vs. 2. 18 ±1.11, p<0.02) compared to S with other headaches types.

## Conclusions

A patient with Mg had a high probability to have a more severe S with increased degree of motor deficit on Rankin disability scale than patients with other types of headache. The relationship between Mg and S is complex and difficult to evaluate and future studies are needed.
